# Differential post-fledging habitat use of Nearctic-Neotropical migratory birds within an urbanized landscape

**DOI:** 10.1186/s40462-018-0132-6

**Published:** 2018-08-20

**Authors:** Zachary S. Ladin, Steffie Van Nieuland, Solny A. Adalsteinsson, Vincent D’Amico, Jacob L. Bowman, Jeffrey J. Buler, Jan M. Baetens, Bernard De Baets, W. Gregory Shriver

**Affiliations:** 1 Department of Entomology and Wildlife Ecology, University of Delaware, Rm. 250 Townsend Hall, 531 South College Avenue, Newark, DE 19716 USA; 2 Department of Data Analysis and Mathematical Modeling, Ghent University, Ghent, Belgium; 3 Tyson Research Center, Washington University in St. Louis, Eureka, Missouri 63025 USA; 4 US Forest Service, Northern Research Station, Newark, DE USA

**Keywords:** Brownian bridge, *Dumetella caroliniensis*, Gray catbird, *Hylocichla mustelina*, Radio telemetry, Wood thrush

## Abstract

**Background:**

Persistent declines in migratory songbird populations continue to motivate research exploring contributing factors to inform conservation efforts. Nearctic-Neotropical migratory species’ population declines have been linked to habitat loss and reductions in habitat quality due to increasing urbanization in areas used throughout the annual cycle. Despite an increase in the number of studies on post-fledging ecology, generally characterized by the period between fledging and dispersal from natal areas or migration, contextual research linking post-fledging survival and habitat use to anthropogenic factors remains limited.

**Methods:**

Here, we examined habitat use of post-fledging habitat-generalist gray catbirds (*Dumetella caroliniensis*), and habitat-specialist wood thrushes (*Hylocichla mustelina*), up to 88 days after fledging within an urbanized landscape. These Neotropical migratory species share many life-history traits, exhibit differential degrees of habitat specialization, and co-occur in urbanized landscapes. Starting from daily movement data, we used time-integrated Brownian bridges to generate probability density functions of each species’ probability of occurrence, and home range among 16 land cover classes including roads from the US Geological Survey National Land Cover Database for each species.

**Results:**

Habitat use differed between pre- and post-independence periods. After controlling for factors that influence habitat use (i.e., pre- or post-independence period, fate (whether individuals survived or not), and land cover class), we found that wood thrushes occupied home ranges containing six times more forest land cover than catbirds. In contrast, catbirds occupied home ranges containing twice the area of roads compared to wood thrushes. Wood thrushes had greater variance for area used (km^2^) among land cover classes within home ranges compared to catbirds. However, once fledglings achieved independence from parents, wood thrushes had lower variance associated with area used compared to catbirds.

**Conclusions:**

Our findings support predictions that habitat-generalist gray catbirds spend more time in developed areas, less time in forest habitat, and use areas with more roads than the forest-specialist wood thrush. We found strong effects of pre- and post-independence periods on all of the response variables we tested. Species-specific habitat use patterns will likely be affected by projected increases in urbanization over the next several decades leading to further reductions in available forest habitat and increased road density, and will have important implications for the ecology and conservation of these birds.

## Background

Organismal movement, habitat use, and interactions with the underlying resources therein, continue to be of particular importance in ecology and conservation [1–4]. Through studying the dynamics of animal movement, we can elucidate important responses to environmental change, especially anthropogenic stressors which can directly influence food availability [5, 6], breeding habitat [7, 8], and cover [9, 10]. Hence, habitat use patterns can be shaped by non-mutually exclusive resource selection interactions and trade-offs in relation to changes in resource availability [11, 12].

Movement data have been used successfully to analyze space and resource use patterns in birds during breeding [13, 14], and specifically, in response to anthropogenic factors [15, 16]. These studies have led to important insights into determining migration routes and migratory connectivity [17–19] which are helping to identify population-limiting factors [20], and improve conservation efforts throughout the annual cycle [21, 22]. Additionally, studies on fledgling movements and habitat use provide key insights into this particularly vulnerable life-stage [23–26].

While more studies are now being conducted to help fill information gaps throughout the annual cycle during migratory [27–30], and over-wintering periods [31–33], there still remain important gaps. One of these critical gaps is related to the post-fledging period [34], which we define for this study as the period after nestlings leave the nest up until departure on fall-migratory flights. Collectively, these research efforts provide more detailed information on how species respond to anthropogenic effects, like urbanization, as well as providing species-specific information which can improve our ability to promote conservation efforts for species like the wood thrush (*Hylocichla mustelina*), which is a forest habitat specialist and Partners in Flight species of continental conservation concern [35].

Given the increasing rate of anthropogenic change and its linkages with observed widespread reductions in habitat availability, quality, and conincident declines in bird populations [36], more research is warranted to help better understand and predict how birds will respond to future urbanized ecosystems. For example, in a recent review and meta-analysis, [37] demonstrated how habitat fragmentation per se, is not only not necessarily negatively correlated with species or community-level responses, but instead may, generally have a positive influence on populations or biological diversity. These findings indicate that complexity associated with land cover changes (beyond simple metrics of fragmentation when accounting for total area of available habitat), must be considered when attempting to tease apart the patterns and processes associated with anthropogenic effects on habitat use, survival, and population trends of species. Indeed, this is being addressed by a growing body of research on bird responses to urbanization [38–41]. However, we must continue to investigate avian responses to urbanization during critical periods in the annual cycle that remain under-studied [34]. Of particular importance is the post-fledging period, during which birds have high mortality rates in general [42–46].

To better improve our understanding of how anthropogenic effects of urbanization can influence habitat use during the post-fledging period, we conducted a comparative study using telemetry-based movement data of individual juvenile wood thrushes and gray catbirds (*Dumetella carolinensis;* hereafter catbird) during the post-fledging period. Although these two species are closely-related phylogenetically [47] and share similar life-history traits [48, 49], they exhibit differential responses to urbanization, in part resulting from their habitat-generalist or –specialist behavior, and currently have opposing annual population trend estimates (catbird: 0.42%, and wood thrush: − 2.77%; [50]. Differing habitat use patterns can then lead to different levels of exposure to examples of potential negative effects from urbanization on birds that include increased risk of mortality due to collisions with buildings or cars [51–53], predation by domestic cats [54–56], pollution [57, 58], and reductions in availability of food and habitat [7, 59–61].

Our objectives were to estimate 1) post-fledging home ranges, and 2) movement metrics of catbirds and wood thrushes during both pre- and post-independence periods, which we define here as ≤ 20 days and > 20 days from leaving the nest [48, 49]. To measure the response of post-fledging habitat use within an urbanized landscape, we examined patterns of habitat use within wood thrush and catbird home ranges during pre- and post-independence periods, and explored their respective use, particularly in relation to forested and roaded habitats. Loss and degradation of high-quality forest habitat, along with increased density of roads are directly related to urbanization and the increasing spread of developed land cover, which can pose myriad threats to wildlife [62, 63], and birds in particular [64, 65]. Through continued study, we can help better understand how species respond (e.g., behavioral aspects of habitat and resource use) within urbanized landscapes. This information is valuable for conserving both urban-tolerant species and urban-avoiding species.

## Methods

### Study area

Our study area in and around Newark, Delaware (39.6837° N, 75.7497° W) and Landenberg, Pennsylvania (39.7778° N, 75.7716° W) is located in the human-dominated eastern mid-Atlantic United States. We tracked catbird and wood thrush fledgling movement using very high frequency (VHF) radio transmitters fitted on individuals that originated from 69 nests within 13 discrete forest fragments in a generally, urbanized landscape [66]. We sampled sites within forest fragments that ranged in area from 5 to 163 ha, and consisted of dominant canopy tree species including *Fagus grandifolia*, *Acer rubrum*, *Quercus* spp*.*, *Liriodendron tulipifera*, and *Liquidambar styraciflua*. Additionally, sites characteristically supported both native and non-native understory woody species including *Lindera benzoin*, *Viburnum* spp., *Clethra alnifolia*, *Rosa multiflora*, *Eleagnus umbellata*, and *Rubus* spp.

### Data collection

During the 2012–2014 breeding seasons (i.e., May–August), we systematically searched 23 forest fragments; 13 of which contained nests of wood thrushes and catbirds. We visually monitored nests every 3–4 days [67]. We banded (with aluminum US Geological Survey bands; Permit number: 23475), and fitted VHF radio-tags to nestling catbirds and wood thrushes 1–3 days prior to fledging [48, 49]. We randomly selected 1 or 2 nestlings from each nest and attached VHF radio-transmitters (weighing < 1 g) ranging in frequencies between 150 and 151 MHz (Blackburn transmitters, Nacogdoches, Texas, USA). We extracted nestlings by hand from nests, attached transmitters using a 1 mm elastic thread fitted with a figure-8 loop harness [68], and then returned nestlings to their nests.

Using hand-held Yagi antennas attached to programmable receivers (Advanced Telemetry Systems, Isanti, Minnesota, USA), we located each individual once per day throughout the study period until they either died, the radio-transmitter battery failed, or they dispersed from the study area. When a bird was successfully located by homing in on individuals, we used global positioning system (GPS) units (± 3-m accuracy) to record location coordinates (latitude and longitude). We identified mortality events when transmitters were recovered containing part or all of a dead individual’s body, blood, or feathers. In some circumstances, where the locations of birds were unable to be determined if an individual was out of range compared to its previous location, we used a roof-mounted omnidirectional antenna on top of a vehicle to search an increasingly larger area beginning at the bird’s previous known location. These searches continued for all missing birds each day and lasted until the bird was located or up to 5 days beyond the estimated date of transmitter battery failure.

### Determining habitat use

To determine the areas that individual birds used, we used a Brownian bridge movement model (BBMM), which is a time-integrated, path-based (as opposed to a point-based) approach to estimate space use. We preferred BBMMs due to recent comparisons among space use estimators suggesting that the incorporation of temporal components leads to more reliable results than traditional kernel-based estimators [69]. Brownian motion (i.e., a random motion of individuals conditioned by a start and end point), is referred to as a Brownian bridge (BB) and is used in the BBMM. In a BB, an individual’s position at any time instance is described by a normal distribution. In between two consecutive fixes, an infinite number of possible paths can be simulated and the (time-independent) marginal probability density function (PDF) at each location can be obtained by averaging these normal distributions over time. The PDF of this so-called time-integrated BB (hereafter referred to as home range) is used in animal movement research to delineate important areas. The latter is realized using the so-called BBMM which relies on the home ranges. More specifically, the BBMM involves the construction of the PDF of a weighted average of home ranges between every two consecutive fixes. The resulting PDF describes the probability density that an animal is positioned at a certain location at an arbitrary moment in time within the considered time interval. It is determined by the registered locations, the time between those locations and the animal’s mobility, which are not accounted for in some alternative approaches [70, 71]. Here, the PDF is constructed for each individual and for each period pre- and post-independence using the analytical solution proposed by [72] using Mathematica (ver. 11.1.1.0). Given that our study focuses on fledglings who are exploring their habitat, the assumption of a random walk in between two fixes seems justified.

From the resulting PDF, the home range can be defined by calculating a threshold so that the subset of pixels having a PDF value in their center that is greater than or equal to this threshold, represents 95% of the volume under the PDF. This subset represents the area with the highest possible PDF values where the individuals are expected to be located during 95% of the studied time interval [73]. Additionally, following censoring individuals that died 1 day after leaving the nest, we categorized the fate of individuals as either survived or died (see [66] for details) independently within pre- and post-independence periods.

**Fig. 1:**
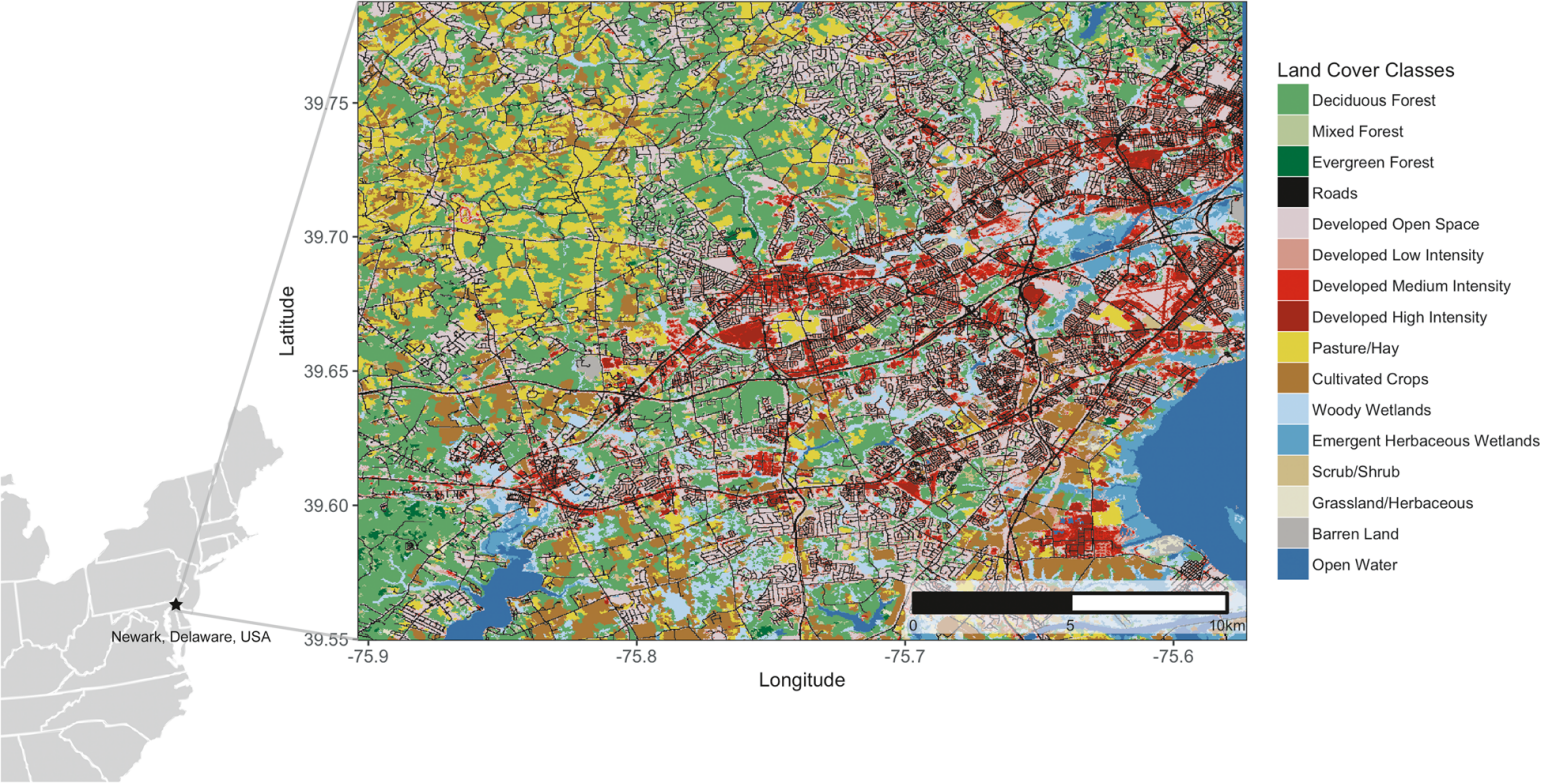
Map of study area in and around Newark, Delaware, United States showing 2011 NLCD and TIGER road layers making 16 land cover classes that were used to compute relative proportions and area (km^2^) within home ranges of post-fledgling gray catbirds and wood thrushes

We used Land Use/Land Cover data from the National Land Cover Database (NLCD; 30 × 30 m resolution; [74]) to quantify relative land cover composition with the areas used by birds during pre- and post-independence periods. These 2011 NLCD data included the following land cover/land use categories: Developed Open Space, Developed Low Intensity, Developed Medium Intensity, Developed High Intensity, Barren Land, Deciduous Forest, Evergreen Forest, Mixed Forest, Scrub/Shrub, Grassland/Herbaceous, Pasture/Hay, Cultivated Crops, Woody Wetlands, Emergent Herbaceous Wetlands, and Open Water [74]. We then embedded roads within the land cover raster using rasterized (30-m resolution) Topologically Integrated Geographic Encoding and Referencing (TIGER) data [75]; Fig. 1).

First, we extracted the total number of cells assigned to each land cover class (including roads as an additional class), and calculated the proportion of each land cover class within the home range for each individual during pre- and post-independent periods. We then weighted these proportions by the PDF value within each cell from within the area of the home range for each individual during pre- and post-independent periods. This resulted in the weighted relative proportion of each land cover class. To determine the respective area (km^2^) of each land cover class, we multiplied the weighted relative proportions (which all summed to 1) by the total area (km^2^) of the home range. We then calculated the mean and standard error for both the weighted relative proportions and area (km^2^) of each land cover class for both species during pre- and post-independence periods. Since none of the individual birds from our study used Grassland/Herbaceous, Emergent Herbaceous Wetland, Barren Land, or Open Water land cover classes, we removed these before performing statistical analyses.

To gain understanding into how underlying movement metrics were related to observed patterns in habitat use within and between species and between pre- and post-independence periods, we computed several movement data metrics. We calculated the mean home range size (km^2^), path length (m), total displacement (m), and net displacement (m). We defined home range size as the size of the 95% CI of the BB. We defined path length as the Euclidean distance between a unique pair of consecutive locations where an individual occurred. We defined total displacement as the sum of distance moved per individual, net displacement as the Euclidean distance between the first and final recorded locations for an individual. We calculated these metrics for each individual during pre- and post-independence periods, and then computed means and variance components.

### Statistical analyses

We conducted a chi-square test to test whether mean relative proportions of land cover classes within home ranges were independent within and between species for both the pre- and post-independence periods. For this test, any land cover classes with counts < 2 were removed. To test null hypotheses that catbirds and wood thrushes responded similarly in their habitat use and movement behavior we used the R package ‘lme4’ [76] to fit generalized linear mixed-effects models. We tested for differences in response variables: land cover area (km^2^) within home ranges to the main effects of and interactions between predictor variables: 1) species, 2) land cover class, and 3) independence status. When significant differences were detected from mixed-effects models (a ≤ 0.05) for predictor variables with more than two factor-levels, we used the ‘multcomp’ package to implement Tukey’s HSD post hoc methods to test for pair-wise differences [77]. In addition, to assess differences within and between species, we compared the coefficient of variation (CV) of land cover area (km^2^) within and among species. Additionally, we computed the difference in CV of land cover area (km^2^) between pre- and post-independence periods by computing a value for delta CV (∆CV = CV_post-independence_ – CV_pre-indpendence_). We also fit generalized linear mixed-effects models for each of the following movement metric response variables (i.e., home range size, path length, total displacement, and net displacement) to the main effect and all interactions of the following predictors: species, fate, and independence status. To account for potential bias due to fledglings dying during the study period, originating from the same nest, and differences in sampling effort among years, we included fate (i.e., died or survived) as a fixed effect, and unique nest ID nested within Year as a random effect within models. To ascertain significant differences, we used parametric bootstrapping (PBtest) to test for differences between full and reduced models (i.e., to test for significant contributions of each independent covariate to variance explained) with the ‘pbkrtest’ package [78]. We tested all data for departures from normality using visual inspection of quantile-quantile residual plots of variables, and all statistical analyses were conducted using R ver. 3.4.3; [79].

## Results

**Fig. 2:**
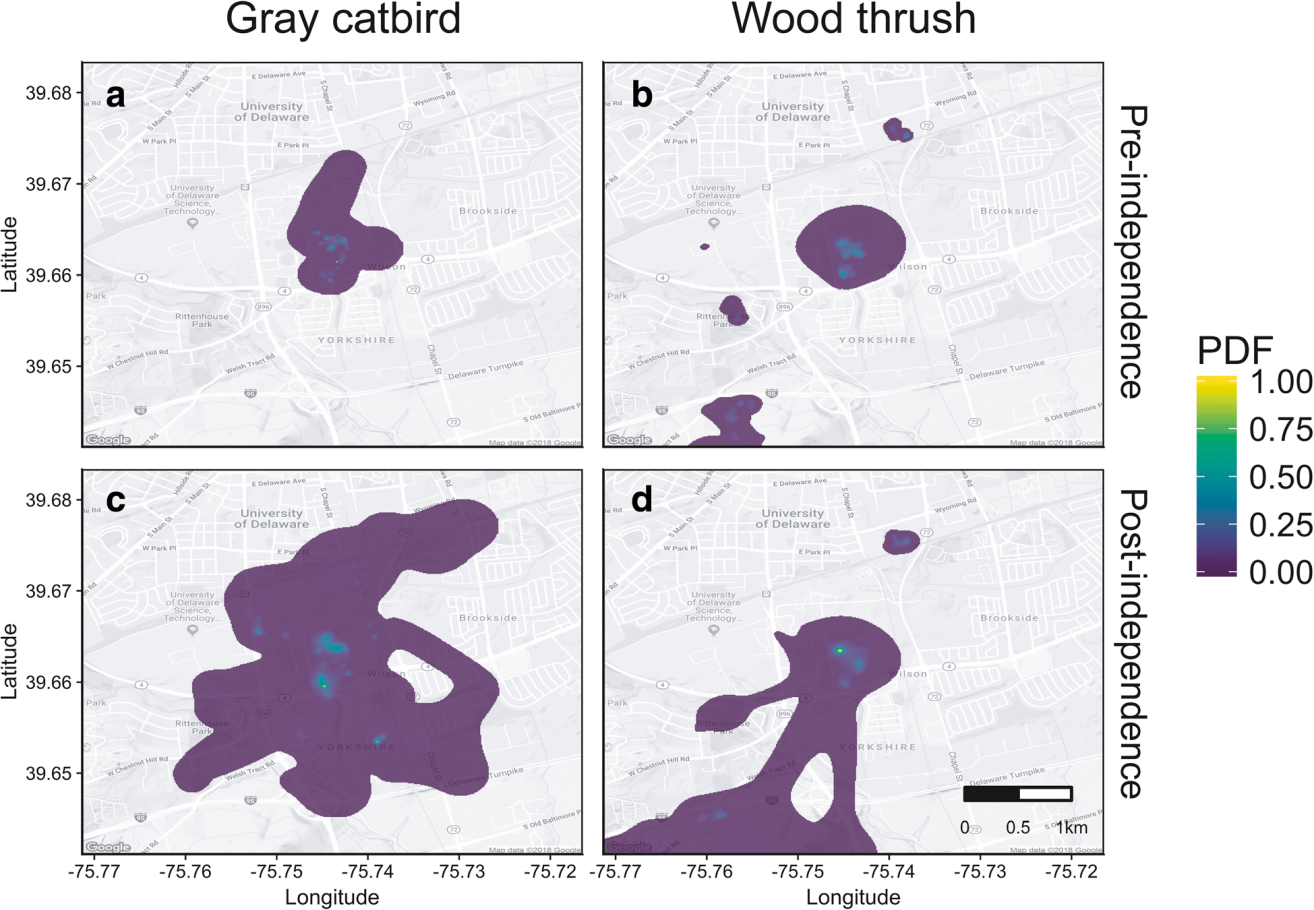
Mean time-integrated Brownian bridge-derived probability density functions (PDF) for fledgling gray catbirds and wood thrushes during pre-independence (**a** and **b**, respectively), and post-independence (**c** and **d**, respectively) periods after fledging during the breeding seasons from 2012 to 2014 in and around Newark, Delaware, USA. All panels show same area in relation to scale bar in panel **d**

We analyzed location data for fledgling catbirds (*n* = 52) and wood thrushes (*n* = 60) that consisted of 4066 unique locations. These data were subset between pre-and post-independence periods and between 44 and 32 catbirds, and 56 and 49 wood thrushes, respectively. On average, individuals were tracked for 41 days (range = 2 to 88 days). Excluding individuals that died on day 1, 69% of catbirds and 86% of wood thrushes survived. From these data, we generated BB-based home ranges for catbirds and wood thrushes during pre- and post-independence periods (see Fig. 2).

**Fig. 3:**
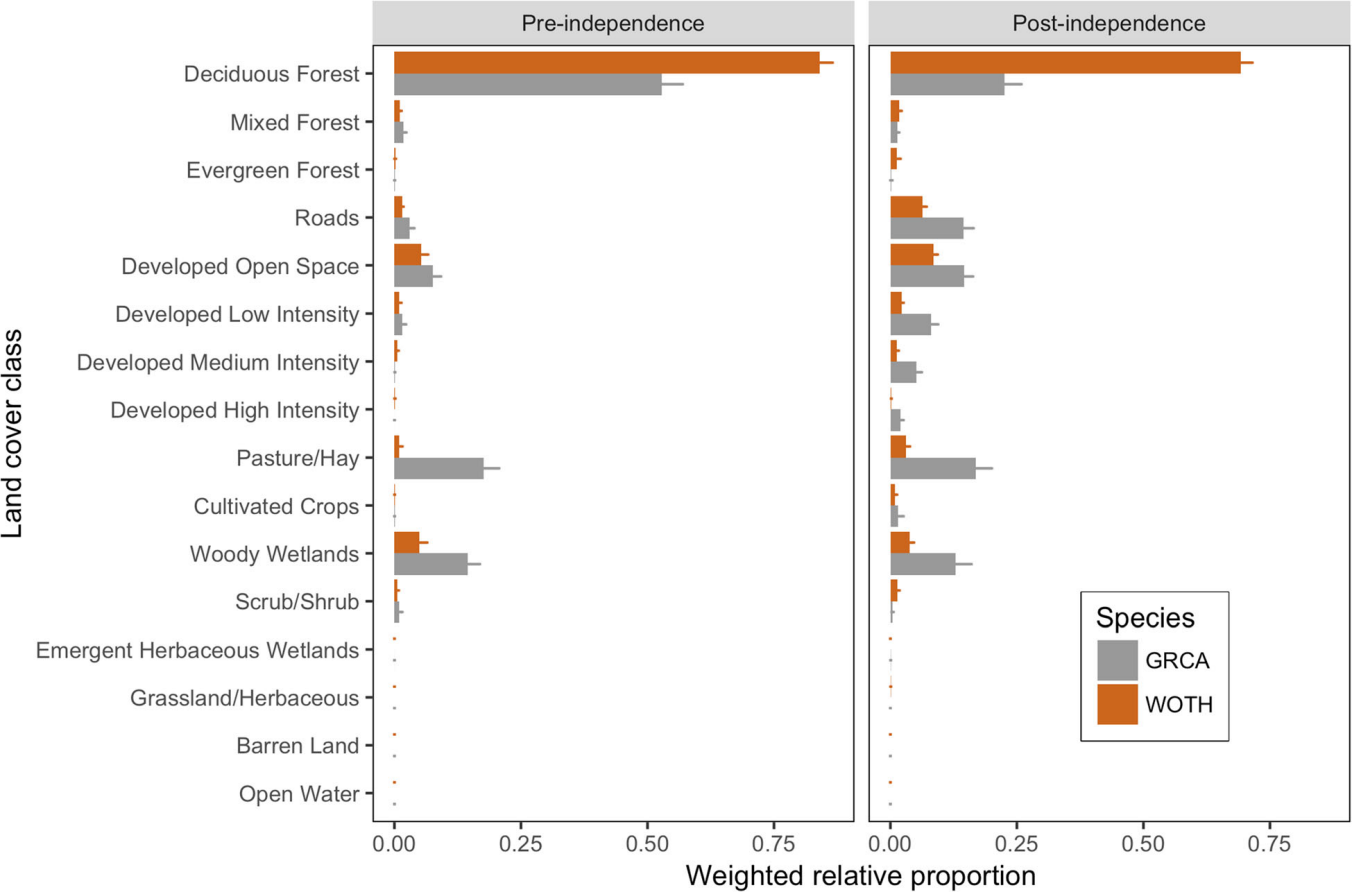
Relative proportions of 16 land cover classes (weighted mean ± SE) within post-fledgling home ranges used by gray catbirds (GRCA; gray bars) and wood thrushes (WOTH; brown bars) during pre- and post-independence periods during the breeding seasons from 2012 to 2014 in and around Newark, Delaware, USA

We found that the relative proportions of land cover classes within home ranges were independent of each other both within and between catbirds and wood thrushes during pre-independence (${\mathcal{X}}^2$ = 70.55, df = 9, *P* <  0.00001) and post-independence (${\mathcal{X}}^2$ = 953.8, df = 12, *P* <  0.00001; Table 1 and Fig. 3).

**Table 1:** Weighted mean relative proportions of habitat use within each National Land Cover Dataset land cover class. Means (and SE) are shown

Land Cover Class	Gray Catbird		Wood Thrush	
	Pre-independence (*N* = 44)	Post-independence (*N* = 32)	Pre-independence (*N* = 56)	Post-independence (*N* = 49)
Deciduous Forest	0.53 (0.04)	0.22 (0.03)	0.85 (0.03)	0.71 (0.02)
Mixed Forest	0.02 (0.01)	0.01 (0.003)	0.01 (0.004)	0.02 (0.004)
Evergreen Forest	0.001 (0.001)	0.002 (0.002)	0.001 (0.001)	0.014 (0.01)
Developed Open Space	0.09 (0.02)	0.2 (0.03)	0.06 (0.01)	0.11 (0.01)
Developed Low Intensity	0.02 (0.01)	0.14 (0.02)	0.01 (0.004)	0.04 (0.01)
Developed Medium Intensity	< 0.01 (< 0.01)	0.09 (0.02)	0.01 (0.003)	0.02 (0.004)
Developed High Intensity	< 0.01 (< 0.01)	0.03 (0.01)	< 0.01 (< 0.01)	< 0.01 (< 0.01)
Pasture/Hay	0.18 (0.03)	0.17 (0.03)	0.01 (0.01)	0.03 (0.01)
Cultivated Crops	< 0.01 (< 0.01)	0.02 (0.01)	< 0.01 (< 0.01)	< 0.01 (< 0.01)
Grassland/Herbaceous	0.00 (0)	0.00 (0)	0.00 (0)	< 0.01 (< 0.01)
Scrub/Shrub	0.01 (0.01)	0.004 (0.002)	0.01 (< 0.01)	0.01 (< 0.01)
Emergent Herbaceous Wetlands	< 0.01 (< 0.01)	< 0.01 (< 0.01)	0.00 (0)	0.00 (0)
Woody Wetlands	0.14 (0.02)	0.12 (0.03)	0.05 (0.02)	0.04 (0.01)
Barren Land	0.00 (0)	0.00 (0)	0.00 (0)	0.00 (0)
Open Water	0.00 (0)	0.00 (0)	0.00 (0)	0.00 (0)

**Fig. 4:**
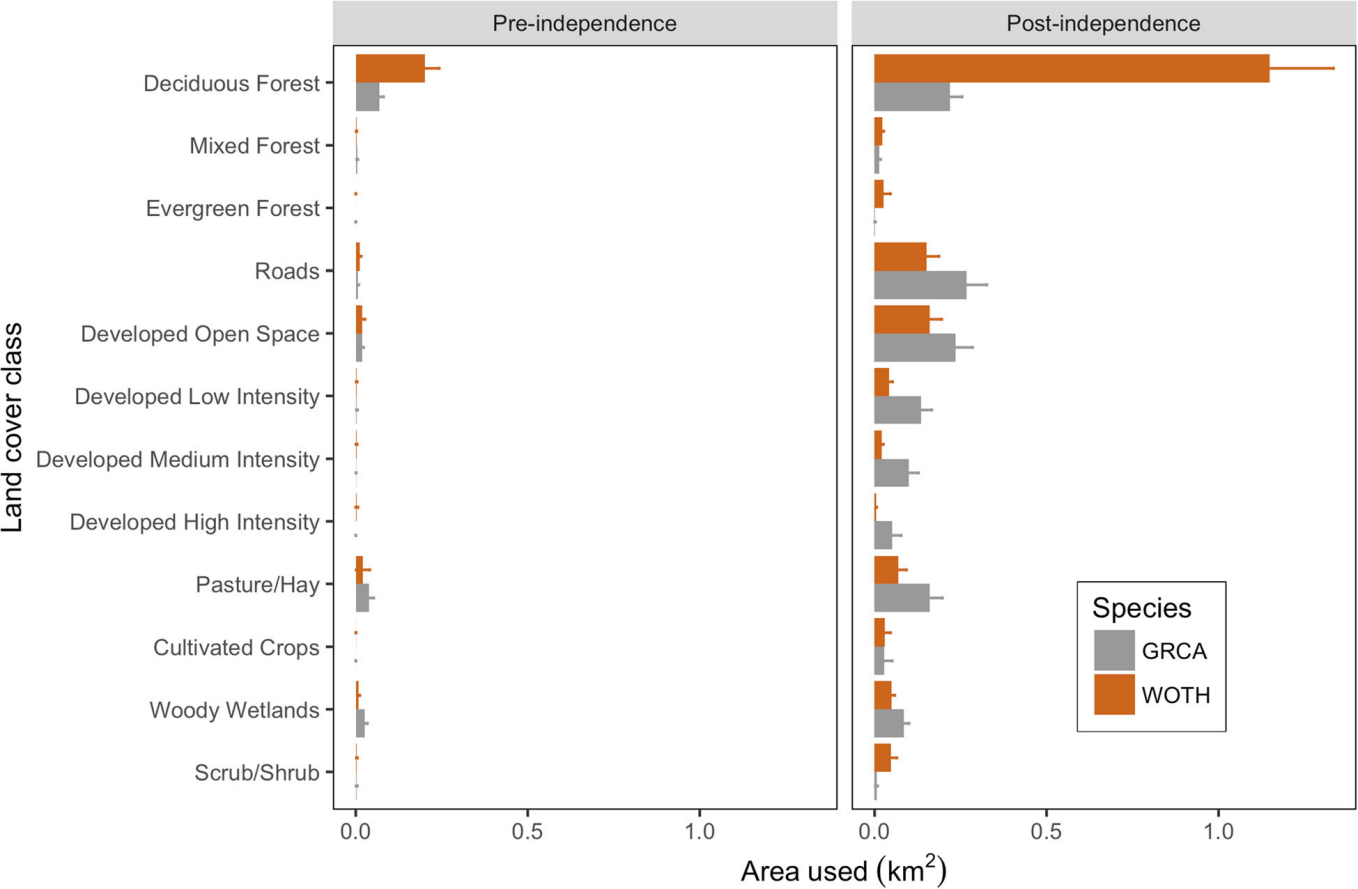
Comparison of the habitat area used (km^2^) in post-fledgling home ranges for 12 land cover classes (weighted mean ± SE) by gray catbirds (GRCA; gray bars) and wood thrushes (WOTH; brown bars) for pre- and post-independence periods during the breeding seasons from 2012 to 2014 in and around Newark, Delaware, USA

We detected significant differences between species among land cover classes (PBtest = 165.1, nsim =1000, *P* <  0.001) that the use of deciduous forest area was five times more frequent by wood thrushes (0.66 ± 0.10 km^2^) compared to catbirds (0.013 ± 0.018 km^2^). Catbird home ranges contained a greater amount of roads (0.12 ± 0.03 km^2^) compared to wood thrushes (0.08 ± 0.02 km^2^; PBtest = 3.82, nsim =1000, *P* <  0.05). Furthermore, we detected a significant post-fledging period × land cover class × species effect (PBtest = 126.6, nsim =1000, *P* <  0.001), where the area of deciduous forest used by wood thrushes was 5.3 times greater than for catbirds during post-independence (t.ratio = − 10.34, *P* <  0.0001; Fig. 4). Catbirds occupied home ranges that contained 1.8 times more road area compared to wood thrushes during the post-independence (t.ratio = 2.77, *P* <  0.04; Fig. 4). Additionally, we detected a significant fate × land cover class × species effect (PBtest = 21.5, nsim =1000, *P* <  0.04), which showed that, of birds that survived, wood thrushes used deciduous forest area 4.9 times more often than catbirds (t.ratio = − 15.2, *P* <  0.0001; Fig. 4). Although we detected a marginally significant post-fledging period × fate × species effect (PBtest = 3.43, nsim =1000, *P* = 0.078), post-hoc pairwise comparisons indicated that catbirds and wood thrushes used areas similarly between fates within pre- and post-fledging periods (t.ratios ranged between − 2.21 and 1.40, *P* > 0.35, in all cases). Moreover, we found no interactive effects of post-fledging period × fate × land cover class × species (PBtest = 15.5, nsim =1000, *P* = 0.19).

**Fig. 5:**
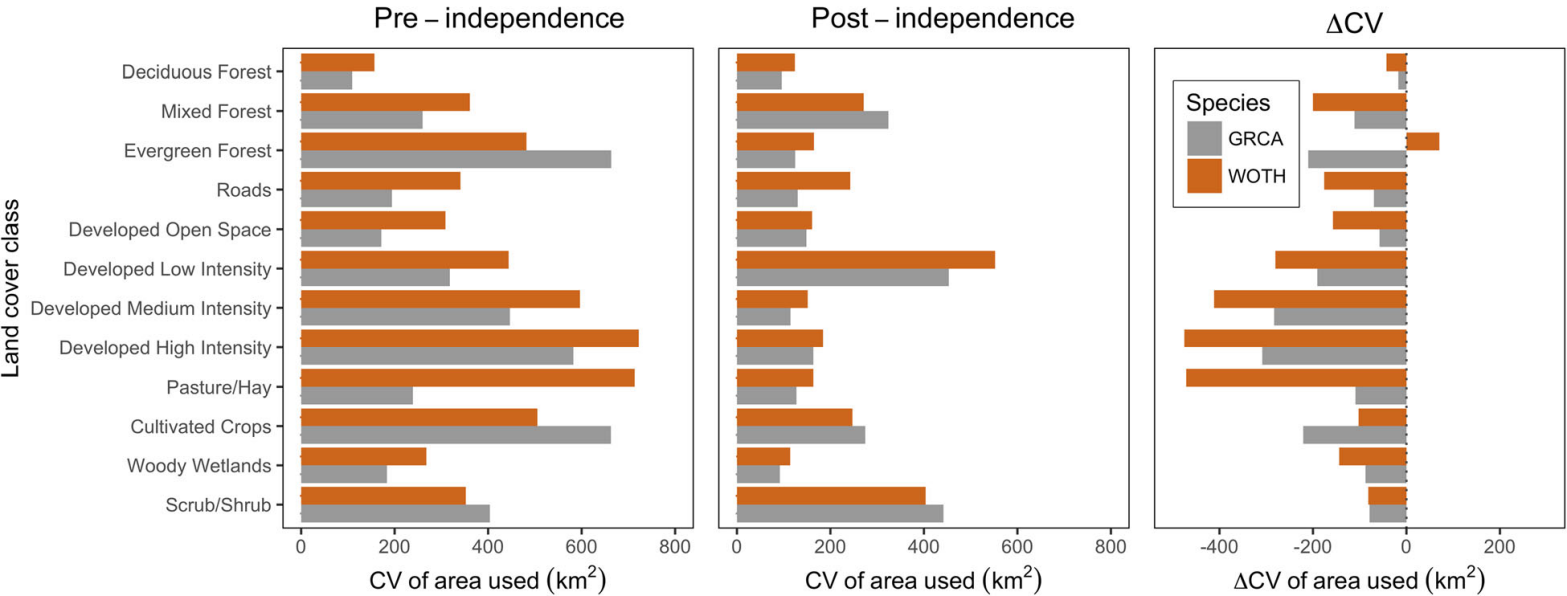
Coefficient of variation (CV) and difference of the coefficient of variation (∆CV) between pre- and post-independence periods of area used (km^2^) within home ranges for 12 land cover classes for gray catbirds (GRCA; gray bars) and wood thrushes (WOTH; brown bars) during the breeding seasons from 2012 to 2014 in and around Newark, Delaware, USA

Wood thrushes had higher CV values for the area used (km^2^) within home ranges than catbirds within all land cover classes except Evergreen Forest, Cultivated Crops, and Scrub/Shrub habitat (Fig. 5). In general, both species had lower CV values during post-independence compared to pre-independence periods (Fig. 5). The computed difference in CV of area used (km^2^) between pre- and post-independence periods (∆CV) showed how the variance decreased for fledglings moving after post-independence compared to pre-independence periods in all cases except for the amount of Evergreen Forest in wood thrush home ranges (Fig. 5). We found that ∆CV was lower in Developed Low, Medium, and High Intensity land cover classes compared to others (Fig. 5). In general, catbirds had greater ∆CV compared to wood thrushes, except for Cultivated Crops and Evergreen Forest land cover classes. These results suggest that both wood thrush and catbird home ranges became more similar, respectively among individuals when after becoming independent of parental care. However, this pattern was more pronounced in the wood thrush.

**Fig. 6:**
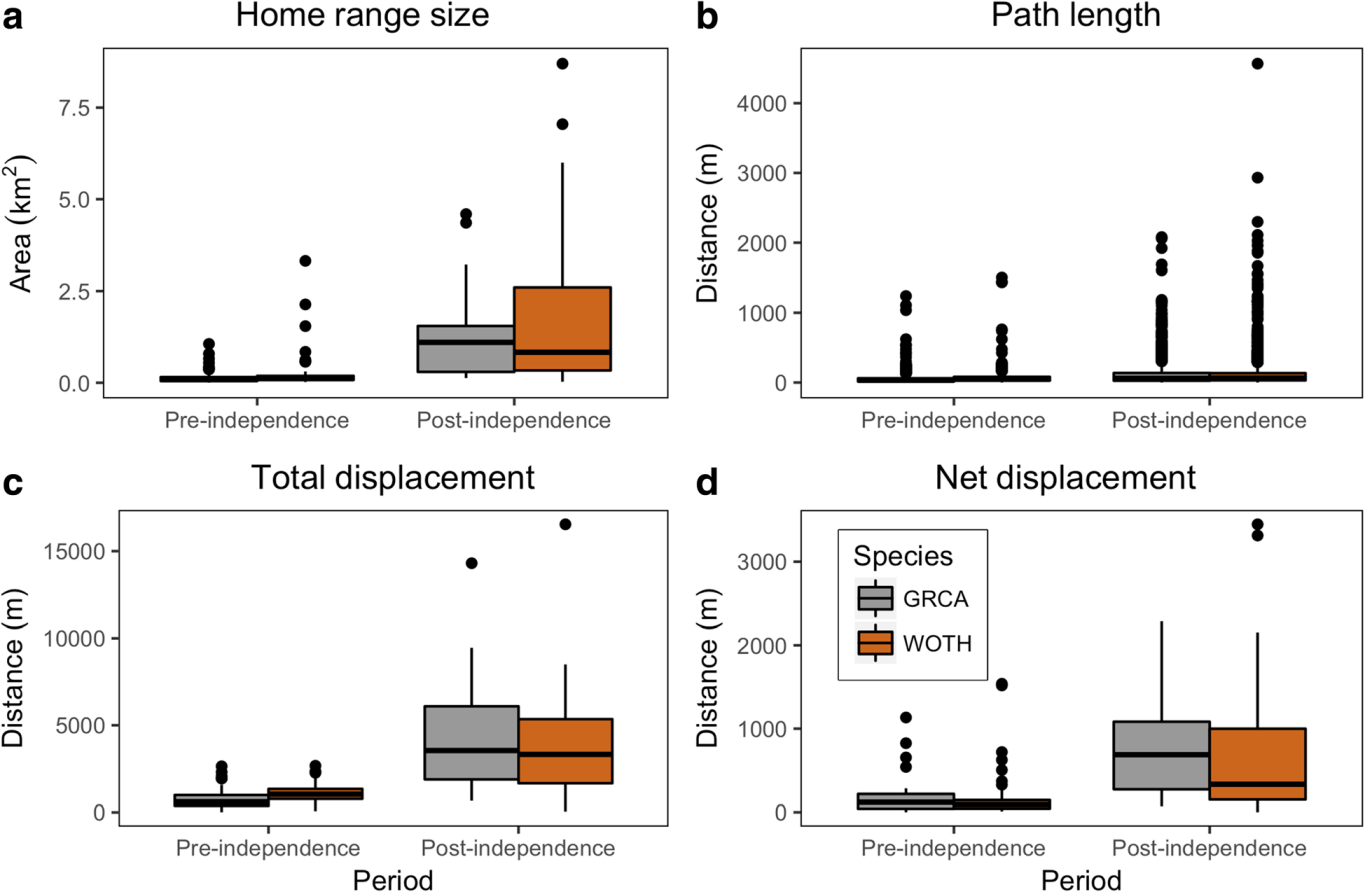
Comparison of pre- and post-independence periods for **a** Home range size (km2), **b** path length (m), **c** total displacement (m), and **d** net displacement. Note differing units and scales on y-axis. Gray catbirds (GRCA) and wood thrushes (WOTH) are shown by gray and brown boxes, respectively

Analyzing the movement metrics underlying habitat use patterns while accounting for all potential interaction terms revealed that movement metrics including home range size, path length, total displacement, and net displacement were all similar between catbirds and wood thrushes (PBtest < 1.73, nsim = 1000, *P* > 0.19 in all cases; Table 2). However, despite a lack of differences between species, we did uncover more generalized patterns between post-fledging periods. For example, we found home range size was 6.9 times greater during post-independence (1.57 ± 0.20 km^2^) compared to pre-independence (0.23 ± 0.04 km^2^) periods (PBtest = 42.3, nsim = 1000, *P* <  0.001; Fig. 6a). Path length differed between pre- and post-fledging periods (PBtest = 109.4, nsim = 1000, P <  0.001), where post-independence path length (132.3 ± 4.5 m) was double the path length during pre-independence (63.5 ± 2.5 m; Fig. 6b). Total displacement moved was greater during post-independence (3989 ± 331 m) than pre-independence (945.4 ± 59 m) periods (PBtest = 86.3, nsimn = 1000, *P* <  0.001; Fig. 6c). Additionally, the net displacement was also greater during post-independence (720.7 ± 80.4 m) than during pre-independence (174.5 ± 25.5 m) periods (PBtest = 44.2, nsim = 1000, *P* <  0.001; Fig. 6d).

**Table 2:** Mean (and SE) of movement metrics of post-fledging gray catbirds and wood thrushes during pre- and post-independence periods from 2012 to 2014 in and around Newark, DE and Landenberg, PA, USA

	Gray Catbird		Wood Thrush	
Metric (units)	Pre-independent	Post-independent	Pre-independent	Post-independent
Home range size (km^2^)	0.17 (0.03)	1.26 (0.22)	0.27 (0.07)	1.77 (0.29)
Path length (m)	57.6 (3.9)	130.6 (6.6)	67.7 (3.3)	133.5 (6.8)
Total displacement (m)	780.6 (93.2)	4244 (534)	1084 (70.9)	3829 (425)
Net displacement (m)	173.3 (31)	759.4 (105)	178.6 (9.8)	696.4 (114)

## Discussion

We demonstrated how two species of post-fledging Nearctic-Neotropical migratory birds with similar evolutionary- and life-history characteristics, yet differing habitat specialization characteristics responded uniquely in their movement and habitat use patterns within an urbanized landscape. In our study, we compared the catbird and wood thrush that are known to exhibit generalist and specialist habitat-selection proclivities, respectively. Our expectations that catbirds would use areas with less forest and more roads compared to wood thrushes, were supported by our results of estimated home ranges of both species in relation to a representative urbanized landscape within our study area, located in the coastal mid-Atlantic United States, embedded within the megalopolis extending from Boston, Massachusetts to Washington, D. C., USA [80, 81].

We found that catbirds used areas with more developed land cover (e.g., more roads in particular) than wood thrushes, and in contrast, wood thrushes used much greater areas of deciduous forest during the entire post-fledging period. These patterns also differed between pre- and post-independence, when fledglings left natal patches, navigating through the urbanized landscape of our study area. These results are suggestive of how species-specific behavior (e.g., tolerant or avoidant behavior of fledglings to areas of higher road density) may have subsequent repercussions on post-fledgling survival found in other recent studies [66]. However, after examining the variance (CV and ∆CV), we were surprised to find that the wood thrush, typically known as a forest interior specialist, showed greater variation in the area of land cover classes comprising individuals’ home ranges compared to catbirds which are generally more of an edge species and habitat generalist. Since we found this pattern to occur in both pre- and post-independence periods, this finding, in part, may be due to the natal areas of sampled birds. Wood thrushes were sampled from a wider set of geographic locations compared to catbirds within our study area, which could give rise to more variation in surrounding habitat types, and hence land cover classes that get included within estimated home ranges. Nonetheless, the reduction we found in CV of area used among land cover classes for both catbirds and wood thrushes during post-independence indicates that home ranges became more similar during this period among individuals.

Our results supported our expectations, and are important in helping to better understand how habitat use during the post-fledging period within the annual cycle, is related to the effects of urbanization on songbird populations [82]. Patterns in basic movement metrics of wood thrushes such as mean net displacement (m) were similar to previous studies of post-fledging movement [83–85]; however, estimates of home range size differed considerably. For example, [83] estimated home range sizes of fledgling wood thrushes ranged between 0.026 and 0.25 km^2^ whereas in our study estimated home range sizes ranged between 0.019 and 8.7 km^2^. In light of the development of improved analysis methods for estimating habitat use [69, 72, 86], observed differences are of course, understandable. Although these ranges overlap at the lower end, the discrepancy in differing upper ranges is likely due to differences in the landscapes and habitat composition between the two studies. Anders et al. [83] study took place in a contiguous mature forest compared to our study area’s fragmented urban landscape. Additionally, it has been recently shown that differences can likely arise when using different estimators of home range size [69]. Moreover, both simulation-based [87], and empirical studies have demonstrated support for how differences in landscape permeability within urban areas can influence bird movement [88–90].

Life within urbanized landscapes is often difficult for breeding birds [36, 91, 92], particularly due to inherent losses of suitable breeding habitat [93]. However, not all species respond to direct and indirect effects of urbanization similarly, likely due to complex and interacting differences among evolutionary- and life-history traits, habitat requirements, and behavioral and phenotypic flexibility [94]. In particular, achieving the habitat requirements (e.g., availability and arrangement) for post-fledging birds to forage, avoid mortality risks, and survive until migratory dispersal can be challenging in urban landscapes [95, 96].

How generalist and specialist species respond to anthropogenic factors (e.g., land cover change related to urbanization) continues to be an important area of ecological and biological study across taxa [97–100] including birds [101–103]. While there are obvious conservation-related motivations for this area of inquiry, improving our fundamental understanding of how habitat generalization and specialization influences community assemblage patterns and species’ ability to adapt to environmental change, in general, remain critical aspects of ecological research [104–107]. Defining if a species is a habitat generalist or specialist remains challenging, however findings from recent studies attempting to better understand how the degree of habitat specialization influence species’ response to land cover change have made notable efforts [105, 107]. For example, after using the continent-wide data set on breeding birds in North America [108] and classified land cover data (250-m resolution), to calculate a species specialization index (SSI) using methods from [104, 106] estimated SSI for gray catbirds (1.10) and wood thrushes (1.48), with a higher SSI value indicating a greater degree of specialization. Given known scale-dependent effects of estimating the degree of habitat specialization [105], we would predict that in the more heavily fragmented, urbanized, and densely-populated New England and mid-Atlantic region of the United States, the difference between catbird and wood thrush SSI may become even more pronounced. In another recent study, [109] found that wood thrush populations at the landscape-scale responded negatively to decreased landscape quality as a function of increased habitat loss and fragmentation (while controlling for amount of habitat) due to urbanization. Collectively, these results support findings from our study, and suggest that future research focusing on local and regional responses of generalist catbirds and more specialized wood thrushes are warranted.

Increased space use within the urban matrix, for example, can be inherently linked to reduced post-fledging survival probability, due to the increase in exposure to both natural and anthropogenic mortality factors [110–112]. Adalsteinsson et al. [66] found that post-fledging survival was higher for wood thrushes than gray catbirds. However, both species experienced an increased risk of mortality due to anthropogenic factors during the post-independence period. This makes sense as it is the period when fledglings are no longer requiring parental provisioning, and are leaving their natal forest patches to explore potentially hostile matrix environments for their first time. Our estimates of habitat use during post-independence corroborate patterns of exposure to anthropogenic mortality factors, as measured by the differences between catbirds and wood thrushes in their use of areas with roads. The resulting number of road crossing events which can be thought of as a proxy for anthropogenic mortality factors, was correlated with respective survival estimates during the post-independence period within our study area [66]. These results provide insight into the mechanisms by which fledglings during post-independence mitigate a naturally incurred higher risk of mortality potentially, due to the increased probability of encountering developed land cover and roads through differential habitat use behaviors. These findings can help lay the groundwork to enable future empirical-based models to help predict how populations of migratory landbirds respond to increasing rates of habitat loss and fragmentation that will likely result from imminent increases in the rate of urbanization over the next several decades.

As increasing rates of urbanization are predicted to increase adverse effects on ecosystems [113], species that depend on critical non-urban breeding habitat such as migratory Neotropical birds are also predicted to experience concomitant declines [114–116]. Given equivocal results as to the effects of urbanization on forest-breeding bird populations from previous studies [109, 117], continued research in this area seems warranted. To manage increasingly urbanized ecosystems, and design landscapes to mitigate potential negative effects, studies ought to continue investigating how species respond, and adapt to both direct and indirect anthropogenic effects of urbanization including loss of habitat, habitat fragmentation, and increased road densities.

## Conclusions

Given predicted increasing rates of urbanization, understanding how direct and indirect linkages between anthropogenic factors and declining migratory songbird species is important. Our study highlights the continuing need to explore the understudied post-fledging period to tease apart how urbanization effects on individuals can play an important role in habitat use. We found that habitat use decisions by two species of post-fledging Nearctic-Neotropical species differing in their degree of habitat specialization, and exhibiting differential tolerance for urbanization responded according to our basic predictions. The wood thrush, a forest interior specialist, used more forest habitat within post-fledging home ranges and avoided roads compared to the more urban-tolerant and generalist species, the gray catbird.

## Abbreviations

∆CV: Delta coefficient of variation
BB: Brownian bridge
BBMM: Brownian bridge movement model
CV: Coefficient of variation
NLCD: National land cover data
PBtest: Parametric bootstrap test
PDF: Probability density function
SSI: Species specialization index
TIGER: Topologically Integrated Geographic Encoding and Referencing
